# All-in-one AAV-mediated *Nrl* gene inactivation rescues retinal degeneration in *Pde6a* mice

**DOI:** 10.1172/jci.insight.178159

**Published:** 2024-12-20

**Authors:** Zhiquan Liu, Siyu Chen, Chien-Hui Lo, Qing Wang, Yang Sun

**Affiliations:** 1 Department of Ophthalmology, Stanford University School of Medicine, Palo Alto, California, USA.; 2 Palo Alto Veterans Administration, Palo Alto, California, USA.

**Keywords:** Ophthalmology, Therapeutics, Gene therapy, Genetic diseases

## Abstract

Retinitis pigmentosa (RP) is a complex group of inherited retinal diseases characterized by progressive death of photoreceptor cells and eventual blindness. *Pde6a*, which encodes a cGMP-specific phosphodiesterase, is a crucial pathogenic gene for autosomal recessive RP (RP43); there is no effective therapy for this form of RP. The compact CRISPR/*Staphylococcus aureus* Cas9 (CRISPR/SaCas9) system, which can be packaged into a single adeno-associated virus (AAV), holds promise for simplifying effective gene therapy. Here, we demonstrated that all-in-one AAV-SaCas9–mediated *Nrl* gene inactivation can efficiently prevent retinal degeneration in a RP mouse model with *Pde6a*^nmf363/nmf363^ mutation. We screened single-guide RNAs capable of efficiently editing the mouse *Nrl* gene in N2a cells and then achieved effective gene editing by using a single AAV to codeliver SaCas9 and an optimal *Nrl*-sg2 into the mouse retina. Excitingly, in vivo inactivation of *Nrl* improved photoreceptor cell survival and rescued retinal function in treated *Pde6a-*deficient mice. Thus, we showed that a practical, gene-independent method, AAV-SaCas9–mediated *Nrl* inactivation, holds promise for future therapeutic applications in patients with RP.

## Abstract 2

The authors used all-in-one AAV-SaCas9-mediated Nrl gene inactivation to efficiently prevent retinal degeneration in Pde6a mice.

## Introduction

Retinitis pigmentosa (RP) comprises a group of inherited disorders in which progressive loss of photoreceptors is marked by initial rod photoreceptor loss followed by cone photoreceptor loss (1, 2). With a prevalence of 1:5,000 to 1:3,000, RP is the most common form of inherited retinal disease, imposing a substantial burden on both individuals and society. This condition ranks among the leading causes of visual impairment and blindness in individuals under 60, affecting over 1.5 million people globally (1, 3). More than 200 pathogenic genes are known to be associated with RP, which restricts the application of traditional gene augmentation therapeutic approaches (3, 4). The intricate nature of the pathogenic genes presents challenges for RP gene therapy and emphasizes the need for treatments independent of specific gene targets.

The gene encoding the phosphodiesterase 6 α subunit (*Pde6a*) belongs to the phosphodiesterase family and plays a crucial role in the retina by regulating the visual signal transduction pathway (5, 6). When *Pde6a* undergoes mutation, the function of phosphodiesterase may be affected, disrupting the visual signal transduction pathway and ultimately causing progressive degeneration and death of photoreceptor cells in the retina (7, 8). Mutations in *Pde6a* contribute to 3%–4% of RP cases; there is no targeted treatment for this blinding disease (1, 8). Affected patients present with night blindness and progressive constriction of their peripheral visual fields while retaining central vision. Loss of rod photoreceptors is followed by loss of cone photoreceptors, causing an irreversible decline in visual acuity that may lead to blindness (8–10).

One potential gene- and mutation-agnostic therapy for RP is knockdown of neural retina leucine zipper (*Nrl*) or nuclear receptor subfamily 2 group E member 3 (*Nr2e3*) in mature retina (11). *Nrl* and *Nr2e3* are members of the basic region-leucine zipper transcription factor family, with *Nrl* acting upstream of *Nr2e3*, that play a crucial role in the development and maintenance of the retina, particularly in the differentiation and homeostasis of rod photoreceptor cells (11, 12). During photoreceptor cells development, *Nrl* functions as a cell fate switch: photoreceptor precursors that express *Nrl* differentiate into rods, while those that do not express it differentiate into cones (11, 13). Based on this principle, an intriguing approach has been proposed — treating retinal degeneration by converting rods into cones through the inhibition of *Nrl* or *Nr2e3* in adult retina (13).

The CRISPR/Cas9 system can induce powerful gene manipulation and offers a versatile genome editing platform with applications in biotechnology and clinical medicine (14–17). Recently, several groups have shown that inhibiting the activity of *Nrl* or *Nr2e3* in the mature retina using adeno-associated virus–delivered (AAV-delivered) *Streptococcus pyogenes* Cas9 (SpCas9) can confer specific cone-like properties on rod cells (18–20). This approach has prevented retinal degeneration in multiple RP mouse models with mutations in *Rho* and *Pde6b* (18–20). Present application of the AAV-delivered SpCas9 system requires packaging SpCas9 and its single-guide RNA (sgRNA) separately into two distinct AAV vectors owing to the constrained cargo capacity of a single AAV vector (18, 19). However, because the use of dual AAV vectors increases the complexity of AAV packaging and delivery, it reduces the efficiency of gene editing, which diminishes the practicality of gene therapy.

In this study, we utilize the state-of-the-art, all-in-one AAV-delivered compact *Staphylococcus aureus* Cas9 (SaCas9) system to inactivate the *Nrl* gene and achieve efficient gene inactivation in the retina of *Pde6a* mutant mice. Our results indicate that inactivation the *Nrl* gene prevents retinal degeneration and preserves cone function in the *Pde6a* mouse model. Therefore, the inactivation of *Nrl* using an all-in-one AAV-delivered SaCas9 system holds the potential to advance future gene therapy applications for patients with RP.

## Results

### Efficient Nrl and Nr2e3 editing in N2a cells by all-in-one AAV-SaCas9 vector.

**Figure 1:**
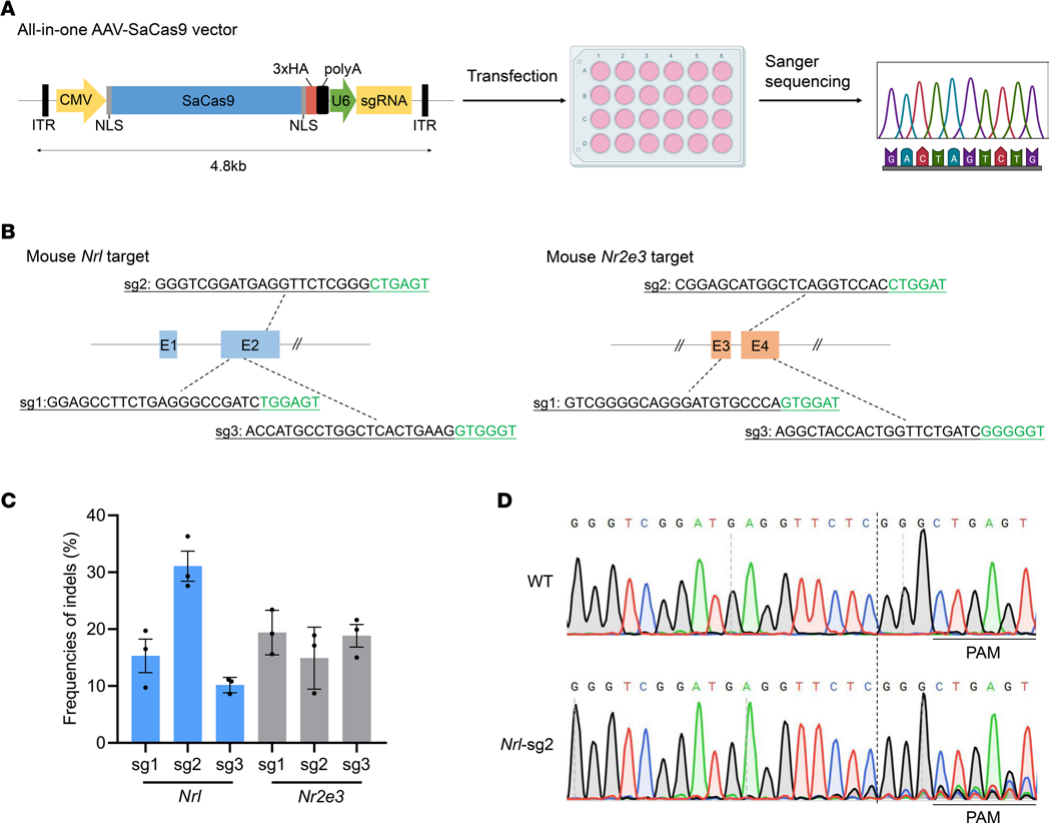
Gene editing of *Nrl*/*Nr2e3* in N2a cells using AAV-SaCas9 vector. (**A**) Workflow for the screening of sgRNAs targeting *Nrl*/*Nr2e3* using the all-in-one AAV-SaCas9 vector in N2a cells (created by BioRender.com). (**B**) Schematic representation of the mouse *Nrl*/*Nr2e3* locus, illustrating the position of the designed sgRNA target. The 21-nt targeted sgRNA sequence is marked in black, and the NNGRRT protospacer adjacent motif sequence is highlighted in green. All sgRNAs were positioned in the coding sequence to disrupt gene function. (**C**) Comparison of the indel efficiency of the tested sgRNAs targeting *Nrl*/*Nr2e3* using the all-in-one AAV-SaCas9 vector in N2a cells. (**D**) Representative Sanger sequencing chromatograms of edited N2a cells at the *Nrl*-sg2 site. The dashed line represents the expected cleavage sites of SaCas9. WT. Data are shown as the mean ± SEM and *n* = 3 biologically independent experiments.

Because the large size of the classical SpCas9 (1,368 aa) system makes it unsuitable for single AAV delivery, a series of compact Cas9 systems, including SaCas9 (1,053 aa) (21), SpaCas9 (1,130 aa) (22), Cje1Cas9 (984 aa) (23), Cje3Cas9 (1,000 aa) (24), and others, have been developed. SaCas9 is the most widely recognized and preferred of these systems for gene therapy (25–28). Here, we employed the cutting-edge, all-in-one AAV-SaCas9 system, deliverable through a single AAV, to target *Nrl* or *Nr2e3* (Figure 1A). We designed 3 SaCas9-targeted sgRNAs for each of the *Nrl* and *Nr2e3* genes (Supplemental Table 1; supplemental material available online with this article; https://doi.org/10.1172/jci.insight.178159DS1). All these sgRNAs feature the optimal NNGRRT (R = A/G) protospacer adjacent motif sequence and predicted low off-target potential (Figure 1B and Supplemental Table 2). To test the efficacy of genome editing with SaCas9, we transfected the 6 SaCas9 vectors targeting *Nrl* or *Nr2e3* into mouse Neuro2a (N2a) cells and determined the editing efficiency through Sanger sequencing (Figure 1A). The SaCas9 system induced insertions and deletions (indels) at all 6 target sites, exhibiting varied editing efficiencies ranging from 10.2% ± 0.8% to 31.1% ± 2.7% (Figure 1C and Supplemental Figure 1). Notably, *Nrl*–single-guide RNA 2 (*Nrl*-sg2) induced the highest editing efficiency, reaching up to 36.3% (Figure 1, C and D). Taken together, these data demonstrate that an all-in-one AAV-SaCas9 system can be used to efficiently edit *Nrl* or *Nr2e3* in N2a cells.

### Efficient Nrl inactivation in the retina of Pde6a mice by an AAV-delivered SaCas9 system.

**Figure 2:**
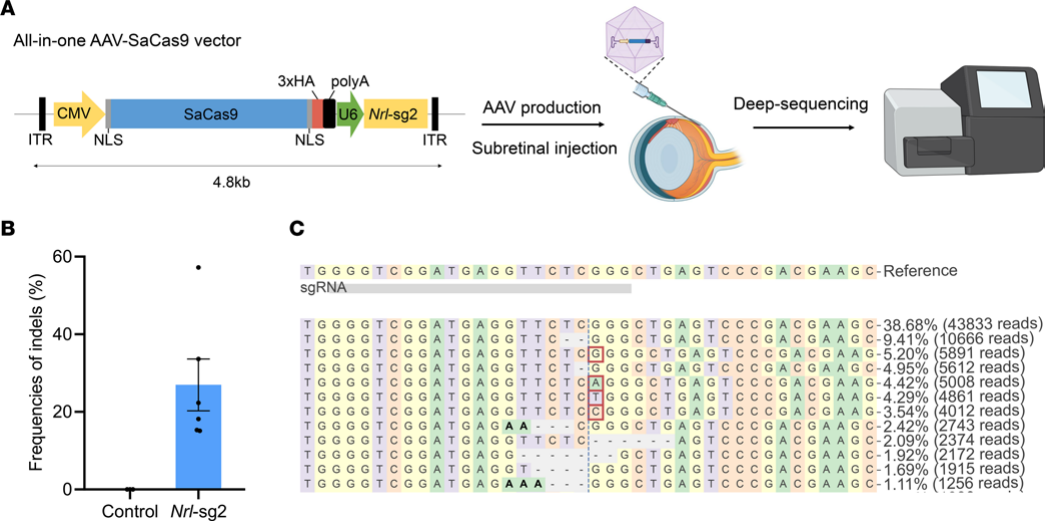
All-in-one AAV-SaCas9–mediated *Nrl* gene inactivation in *Pde6a* mice. (**A**) Workflow for AAV-SaCas9 production, subretinal injection, and efficiency detection by deep sequencing (created by BioRender.com). (**B**) Editing efficiency of the *Pde6a* mouse retina at the *Nrl*-sg2 site, as determined by deep sequencing. Control, *n* = 3; *Nrl*-sg2, *n* = 6. (**C**) Representative deep-sequencing results of edited mouse retina at the *Nrl*-sg2 site (read percentages >1%). Substitutions are shown in bold font. Red rectangles highlight inserted sequences. Horizontal dashed lines indicate deleted sequences. The vertical dashed line indicates the predicted SaCas9 cleavage site.

The *Pde6a*^nmf363/nmf363^ mouse (hereafter referred to as *Pde6a* mice), which carries a missense mutation (c.2009A>G, p.D670G) in the *Pde6a* gene and exhibits moderate photoreceptor degeneration, has been used to model *Pde6a*-related RP (8, 29). Due to the maximal editing efficiency of *Nrl*-sg2, we selected it for in vivo testing in *Pde6a* mice. The combined size of SaCas9 and *Nrl*-sg2 is small enough for packaging into a single AAV vector (Figure 2A). Because the AAV2.NN serotype, a derivative of AAV2 developed through in vivo selection, demonstrates improved retinal and cellular transduction properties (30, 31), we used it to deliver the all-in-one AAV-SaCas9 targeting *Nrl*-sg2 construct to *Pde6a* mice by subretinal injection at P7 (Figure 2A). At P60, retinal tissue DNA was extracted from injected mice for deep sequencing to determine gene editing efficiency (Figure 2A). *Nrl* editing was efficient in all 4 tested mice: efficiencies ranged from 15.1% to 57.2% (Figure 2B). Deep-sequencing results also indicated that SaCas9 primarily induced small insertions or deletions of 1–3 base pairs in the *Nrl*-sg2 site (Figure 2C). To verify whether the *Nrl* gene inactivation effectively reduced NRL protein production, we conducted Western blot analysis. The results revealed that the NRL protein levels in the *Nrl*-sg2 group decreased by approximately 40% as compared with that in the control group (Supplemental Figure 2). We also performed quantitative real-time PCR (qPCR) to measure the relative expression levels of rod-specific and cone-specific genes following *Nrl* inactivation. The results showed that, compared with the control group, some rod-specific genes were downregulated while cone-specific genes were upregulated in the *Nrl*-sg2 group (Supplemental Figure 3). In addition, compared with the nonedited control group, no apparent off-target indels at potential off-target sites were observed in the edited mice (Supplemental Figure 4). The result was consistent with that from previous reports highlighting SaCas9 as a genome editing system with high specificity (21, 32–34). Collectively, these data demonstrated efficient inactivation of *Nrl* in *Pde6a* mice and validate the potential of the SaCas9 system for in vivo editing through single AAV delivery.

### Rescue of retinal photoreceptor degeneration in Pde6a mice by Nrl inactivation.

**Figure 3:**
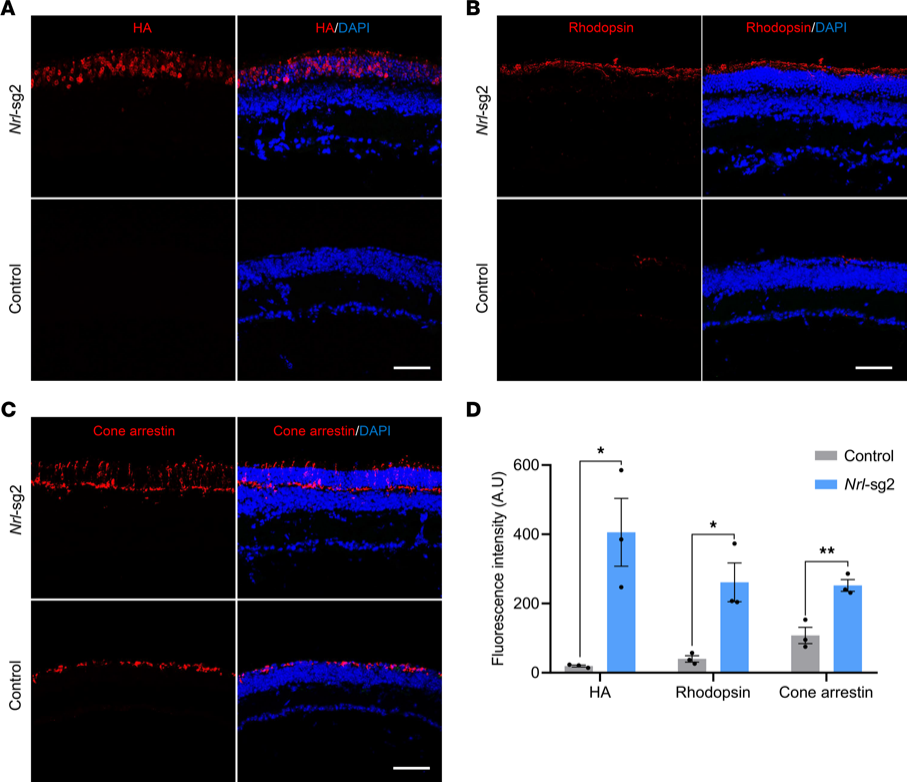
Preservation of retinal photoreceptors in *Pde6a* mice by *Nrl* gene inactivation. (**A**–**C**) Representative immunofluorescence images of retinal sections in *Nrl*-edited or *Pde6a* control mice at P60. HA (**A**), rhodopsin (**B**) and cone arrestin (**C**) indicate SaCas9 expression, rod photoreceptors, and cone photoreceptors, respectively. Scale bar, 50 μm. (**D**) Quantification of the fluorescence intensities of HA, rhodopsin, and cone arrestin in *Nrl*-edited or *Pde6a* control mice at P60. Data are shown as the mean ± SEM and *n* = 3 biologically independent experiments. All *P* values were calculated by 2-sided *t* tests. *P < 0.05, **P < 0.01.

Encouraged by the deep-sequencing results, we next investigated whether *Nrl* editing mediated by the all-in-one AAV-SaCas9 system could preserve retinal photoreceptors in *Pde6a* mice. Retinas from P60 mice were frozen-sectioned and immunostained using antibodies against HA tag (indicative of SaCas9, as shown in Figure 2A), rhodopsin (a marker for rod photoreceptors), and cone arrestin (a marker for cone photoreceptors). Immunoblotting for HA tag confirmed successful delivery and expression of SaCas9 by retinal photoreceptors after subretinal injection of AAV (Figure 3A). Importantly, in comparison to the relatively weak signals of rhodopsin and cone arrestin in *Pde6a* control retinas, staining for these phototransduction-relevant proteins was more robust while continuing to be appropriately localized in retinas treated with *Nrl*-sg2 (Figure 3, B and C). The quantitative analysis of fluorescence intensity substantiated the effective preservation of retinal photoreceptor cells in the *Nrl*-edited retinas, in contrast with the degenerative loss observed in the control group (Figure 3D). The immunostaining of 2 other cone-specific markers, S-opsin and M-opsin, showed similar results (Supplemental Figure 5). We also performed immunostaining on the retinas of P30 mice. The results showed that, although the retinas of *Pde6a* mice had not yet fully degenerated at P30, the treatment group exhibited a certain degree of slowed retinal degeneration (Supplemental Figure 6). Overall, these results strongly indicated that AAV-SaCas9–mediated *Nrl* inactivation effectively prevents retinal photoreceptor degeneration in *Pde6a* mice.

### Rescue of retinal function in Pde6a mice by Nrl inactivation.

**Figure 4:**
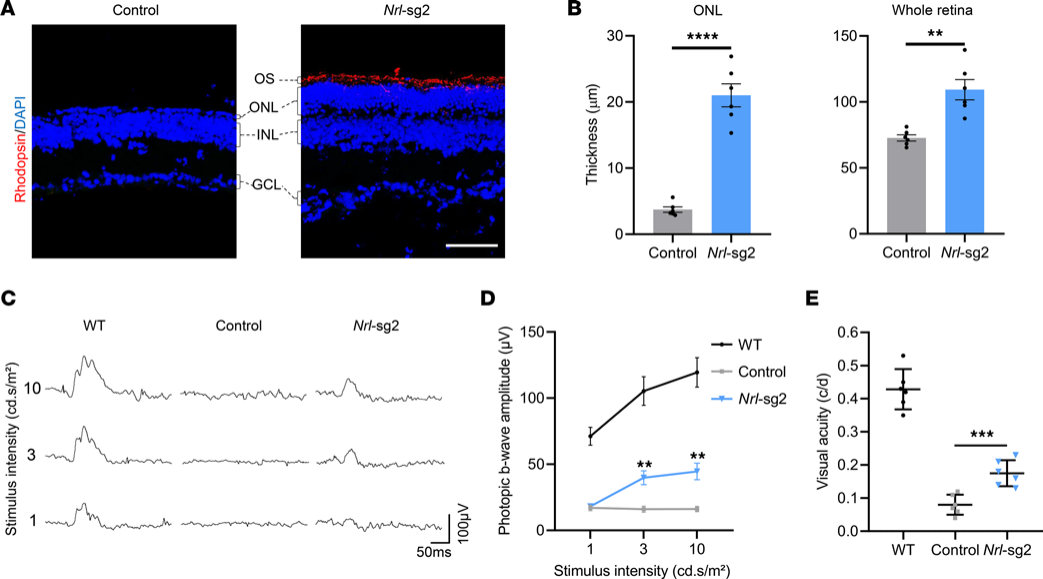
Preservation of retinal function in *Pde6a* mice by *Nrl* gene inactivation. (**A**) Representative images of retinal structure in *Nrl*-edited or *Pde6a* control mice at P60. OS, outer segments; ONL, outer nuclear layer; INL, inner nuclear layer; GCL, ganglion cell layer. (**B**) Quantification of ONL and whole retina thickness in DAPI nuclei–stained retinal sections in *Nrl*-edited or *Pde6a* control mice at P60. (**C**) Representative photopic ERG responses of WT, *Pde6a* control, or *Nrl*-edited mice at P60. The light stimulus intensities were 1, 3, and 10 cd.s/m^2^. (**D**) Quantification of photopic ERG b-wave amplitudes from WT, *Pde6a* control, or *Nrl*-edited mice at P60. (**E**) Quantification of visual acuity in WT, *Pde6a* control, or *Nrl*-edited mice by OKR testing. Data are shown as the mean ± SEM and *n* = 6 biologically independent experiments. All *P* values were calculated by 2-sided *t* tests. **P < 0.01, ***P < 0.001, ****P < 0.0001.

To further examine the effectiveness of *Nrl* inactivation for gene therapy, we assessed retinal functions in both treated and control *Pde6a* mice. Previous reports have indicated that this *Pde6a* RP mouse model exhibits severe photoreceptor degeneration and impaired retinal morphology and function 1 month after birth (5, 29). As shown in Figure 4A, the *Pde6a* control retinas exhibited sparsely distributed photoreceptor cell nuclei in the outer nuclear layer (ONL) at P60, whereas the *Nrl*-sg2 treated retinas displayed a substantially thicker ONL. The quantitative assay of retinal sections in the *Nrl*-sg2 group revealed that ONL thickness measured 23.1 ± 2.6 μm and whole retina measured thickness 120.2 ± 11.4 μm, representing 6.7-fold and 1.6-fold increases compared with those in the control group (Figure 4B). To assess whether the morphological preservation of retina supported visual functional preservation, we used electroretinography (ERG) and optokinetic tracking response (OKR) to measure the electrical activity of photoreceptors and visual acuity at P60. The *Nrl*-sg2 group exhibited marked improvement of the photopic ERG b-wave compared with the control group but no improvement of the scotopic ERG b-wave, indicating preserved cone function (Figure 4, C and D, and Supplemental Figure 7). OKR testing revealed higher visual acuity in the *Nrl*-sg2 group than in the control group (Figure 4E). Taken together, these data suggested that in vivo *Nrl* inactivation efficiently restored retinal function in *Pde6a* mice.

## Discussion

CRISPR-mediated gene inactivation holds great promise for the permanent effective treatment of many genetic diseases, particularly inherited retinal disorders (35, 36). The eye offers an especially advantageous target for gene therapy because it is a relatively independent and immune-privileged organ, which provides easy access for the administration, delivery, and observation of therapeutic effects (37, 38). In this study, we utilized a compact SaCas9 system to efficiently edit *Nrl* in vivo and rescue retinal structure and function in a *Pde6a* RP mouse model. The compact size allows SaCas9 and its sgRNA to be integrated into a single AAV vector for in vivo delivery, streamlining AAV packaging and enhancing the feasibility, practicality, and usability of gene therapy. Notably, the *Nrl* inactivation treatment preserved retinal photoreceptors and ONL thickness and enhanced visual function compared with the control group. In addition, we administered AAV injections to P30 mice, by which time the retinas of *Pde6a* mice had mostly degenerated. The results revealed no obvious therapeutic effect of the injection at P30, suggesting that the optimal treatment window is preferably in the early stage (Supplemental Figure 8).

The present results demonstrate that AAV-SaCas9–mediated postnatal *Nrl* inactivation can effectively rescue retinal structure and function in a RP mouse model of *Pde6a*^nmf363/nmf363^. Together with previously reported therapeutic studies of mice with mutations in the *Pde6b* and *Rho* genes, these findings further validate the *Nrl* inactivation approach as a feasible gene- and mutation-agonistic treatment for RP (18–20). Although numerous gene supplementation or gene-editing approaches have been developed or are in development to treat RP, they often target specific genes or mutations, limiting the scope and practicality of clinical treatment (38). In contrast, the universality of the *Nrl* editing method, which is independent of specific genes or mutations, holds promise for application in a broad range of patients with RP. The current mouse studies indicate that blocking the *Nrl*/*Nr2e3* pathway may reprogram rods into cone-like photoreceptors, potentially preventing the degeneration of retinal rods and cones (11, 18). However, more detailed research is required to elucidate the specific mechanisms of *Nrl* editing in treating RP. Further investigation into the feasibility of *Nrl* gene inactivation therapy in humans is also necessary, considering the potential differences between humans and mice.

However, using the method of inactivating *Nrl* for clinical treatment still faces several challenges. One of these is that the approach requires AAV injections at an early stage of retinal degeneration, as it shows no obvious effects in late stage, posing a challenge for its application in clinical patients. Another concern is the potential side effects that may arise from *Nrl* inactivation. It is well known that *Nrl* is an important transcriptional factor during early retina development, and congenital mutations in the *Nrl* gene are one of the causes of RP (11). However, many studies have shown that inactivating *Nrl* in adults can also prevent retinal degeneration (13, 18–20). One potential explanation is that the downstream effects of inactivating *Nrl* in adult retinas are not the same as those of congenital *Nrl* inactivation. Nevertheless, the potential side effects of *Nrl* inactivation in adult retinas still need to be further investigated in the future. Additionally, since *Nr2e3* functions as a downstream transcription factor of *Nrl*, theoretically, targeting *Nr2e3* could result in fewer potential side effects compared with targeting *Nrl* directly. Like *Nrl*, several studies have demonstrated that disrupting *Nr2e3* can preserve cone morphology and function in mouse models of retinal degeneration (19, 39, 40).

In addition, it is important to note that *Nrl* editing only partially rescued the phenotype of RP mice. Exploring synergistic combinations with other treatment methods is still needed to further enhance the therapeutic effects, such as coediting *Nrl* and *Nr2e3* and codelivering additional neuroprotective genes. However, because these methods exceed the current packaging limitation of a single AAV-SaCas9 system, they require consideration of reducing the size of current Cas9 components or utilizing multiple AAVs for delivery. A viable approach might be to leverage recently reported hypercompact CRISPR- or transposon-encoded RNA-guided nucleases systems, such as Un1Cas12f1 (529 aa) (41), AsCas12f1 (422 aa) (42, 43), TnpB (~400 aa) (44), and IscB (~500 aa) (45, 46). These innovative RNA-guided nucleases are roughly half the size of the current SaCas9, rendering them suitable for multi-sgRNA editing or versatile applications.

In summary, we utilized a compact SaCas9 system that induced efficient *Nrl* editing in vivo by all-in-one AAV delivery. The treated RP mice with *Pde6a* mutation exhibited efficient restoration of retinal morphology and visual function. We anticipate that AAV-SaCas9–mediated *Nrl* inactivation holds promise as a therapeutic method for the future treatment of RP.

## Methods

### Sex as a biological variable.

Our study examined male and female animals, and similar findings were reported for both sexes.

### Animals.

*Pde6a*^nmf363/nmf363^ mice (29) were a gift from Vinit B. Mahajan (Department of Ophthalmology, Stanford University). Animals were housed under a 12-hour light/12-hour dark cycle with access to water and food.

### Plasmid construction.

The AAV-SaCas9 plasmid was obtained from Addgene (plasmid 61591). The sgRNAs targeting Nrl/Nr2e3 were designed by Cas-Designer (47). All sgRNA oligos were synthesized by Azenta Life Sciences and then annealed and ligated into the *BsaI*-digested AAV-SaCas9 plasmid. The sequences of sgRNA oligos are listed in the Supplemental Table 1.

### Cell culture and transfection.

The N2a cell line (ATCC, CCL-131) was cultured in Dulbecco’s Modified Eagle’s Medium (Corning, 10013CV) supplemented with 10% fetal bovine serum and incubated at 37°C in an atmosphere of 5% CO_2_. The cells were seeded in 24-well plates and transfected using PolyJet In Vitro DNA Transfection Reagent (SignaGen Laboratories, SL100688) according to the manufacturer’s instructions. Briefly, 1.5 μL PolyJet reagent with 500 ng AAV-SaCas9 plasmid was added to each well. After 72 hours, the transfected cells were lysed using the One Step Mouse Genotyping Kit (Vazyme, PD101) according to the manufacturer’s instructions. The primers used to amplify target sequences are listed in Supplemental Table 3. Sanger sequencing results were analyzed by TIDE (48).

### AAV production and injection.

The AAV-SaCas9–*Nrl*-sg2 was packaged with serotype AAV2.NN (30) and generated by the AAVnerGene. The titer of the produced AAV was 2 × 1013 genome copies/mL (GC/mL). For AAV delivery, *Pde6a* mice received approximately 1 × 10^10^ GC AAV per eye after dilution via subretinal injection at P7. Mice were anesthetized with ketamine, and pupils were dilated by 1% topical tropicamide. Subretinal injections were administered under an ophthalmic surgical microscope with Picospritzer III microinjection system and a custom-crafted glass micropipette. Approximately 0.5 μL AAV was injected into the subretinal space through a small scleral incision.

### Targeted deep DNA sequencing.

Top 10 potential off-target sites for *Nrl*-sg2 were predicted using Cas-OFFinder (49). Genomic DNA was extracted from injected mouse retinas at P60 using the FastPure Cell/Tissue DNA Isolation Mini Kit (Vazyme, DC102), according to the manufacturer’s protocols. Deep-sequencing primers were designed with generic adapters, and PCR was performed using Phusion High-Fidelity DNA Polymerase (Thermo Scientific, F530L). Targeted deep DNA sequencing was conducted using the Amplicon-EZ sequencing service from Azenta Life Sciences. More than 50,000 reads were generated with each sample using the Illumina platform. Data analysis was performed with CRISPResso2 (50). The primers used to amplify on-target and off-target sequences are listed in Supplemental Tables 3 and 4.

### Western blot analysis.

For Western blot analysis, the mouse retinas were dissected and homogenized in 200 μL RIPA Lysis Buffer (MilliporeSigma, 20-188) supplemented with a protease inhibitor cocktail (Thermo Scientific, 78430). The protein concentrations were measured with the Pierce BCA Protein Assay Kit (Thermo Scientific, 23227). Anti-NRL antibody (Proteintech, 17388-1-AP, 1:500) and anti-Alpha Tubulin antibody (Proteintech, 11224-1-AP, 1:5000) were used as primary antibody and internal control, respectively. Signals were acquired by direct measurement of chemiluminescence using a digital camera (Amersham Imager 600).

### qPCR analysis.

Total RNA was extracted from the mouse retinas using the Quick-RNA Miniprep Plus Kit (Zymo Research, R1058) according to the manufacturer’s instructions. The cDNA was synthesized with the HiScript II 1st Strand cDNA Synthesis Kit (Vazyme, R212). Primers used for qPCR are listed in Supplemental Table 5. The qPCR was performed using the BioEasy SYBR Green I real-time PCR kit with the Bio-Rad CFX Opus 384 multicolor real-time PCR detection system. The relative gene expression normalized to *Gapdh* was determined by the 2^–ΔΔCT^ method. All gene expression data experiments were performed 3 times, and data are expressed as the mean ± SEM.

### Immunofluorescence analysis.

Mice were euthanized using CO_2_, and eyeballs were enucleated and fixed in 4% PFA. Retinas were carefully dissected and subjected to a sucrose gradient series (5%, 15%, 30% sucrose). The retinas were then embedded in OCT compound and stored at –80°C. Cryosections of 15 mm thickness were prepared using a Leica CM1950 cryostat (Leica Biosystems). The retinal cryosections were rinsed in PBS, blocked in a solution comprising 0.1% Triton X-100 and 3% BSA in PBS for 30 minutes at room temperature, and then incubated overnight at 4°C with primary antibodies diluted in the blocking buffer within a humidified chamber. Following 3 PBS washes with 0.1% Triton X-100, sections were exposed to secondary antibodies for 2 hours. DAPI was used to counterstain cell nuclei for 10 minutes. Slides were then mounted using Fluoromount-G mounting medium (Southern Biotech) and covered with a coverslip. The following antibodies were used: rabbit anti-HA tag (Cell Signaling, 3724, 1:500), mouse anti-Rhodopsin (Abcam, ab5417, 1:500), rabbit anti-Cone arrestin (Millipore, AB15282, 1:500), rabbit anti–S-opsin (Millipore, AB5407, 1:500), and rabbit anti-M-opsin (Millipore, AB5405, 1:500). The Alexa Fluor 555–conjugated anti-mouse or rabbit IgG (Invitrogen, 1:500) was used as a secondary antibody. All images of retinal sections were captured by a Zeiss LSM880 inverted confocal microscope. The fluorescence intensities were quantified by ImageJ software (NIH).

### ERG.

Mice were given 12 hours to adapt to the dark before ERG recordings were taken, during which they were anesthetized by ketamine based on their body weight (0.08 mg ketamine/g + 0.01 mg xylazine), and their pupils were dilated by 1% tropicamide. The ERG was performed with an ERG stimulator (Celeris, Diagnosys LLC) according to the manufacturer’s instructions. For scotopic ERG, mice were stimulated with flashes of 0.01, 0.1, and 1 cd.s/m^2^ light intensity. For photopic ERG, mice were given 10 minutes to adapt to the light and then stimulated with flashes of 1, 3, and 10 cd.s/m^2^ light intensity.

### OKR.

The detailed procedure has been previously published (51, 52). Briefly, the OKR) was assessed using the OptoMotry system (CerebralMechanics Inc.), a virtual-reality platform designed to swiftly quantify visuomotor behavior. Mice were positioned on a central platform surrounded by 4 computer monitors equipped with a video camera positioned overhead to record the animal’s movements. A rotating cylinder displaying vertical sine-wave gratings was projected onto the monitors. The OptoMotry software controlled the spatial frequency of the grating to assess the spatial acuity (cycle/degree) of the mouse being tested. The mouse’s tracking of the gratings was reflected through head and neck movements. The maximum spatial frequency of each eye was determined by gradually increasing the spatial frequency of the grating until the mouse ceased tracking.

### Statistics.

All data are expressed as mean ± SEM of at least 3 individual determinations for all experiments. Data were analyzed by 2-tailed Student’s *t* test via GraphPad prism software 8.0.1. A probability value smaller than 0.05 (*P* < 0.05) was considered as statistically significant.

### Study approval.

All animal experimental procedures were performed in compliance with animal protocols approved by the IACUC at Stanford University School of Medicine (protocol ID 32223).

### Data availability.

Deep sequencing data have been deposited in the National Center for Biotechnology Information Sequence Read Archive database (https://www.ncbi.nlm.nih.gov/bioproject/?term=PRJNA1121624). Values for all data points in graphs are reported in the a data values file.

## Author contributions

ZL conceived and designed the experiments. ZL and SC performed the experiments and analyzed the data. CHL and QW contributed reagents, materials, and/or analysis tools. ZL wrote the paper. YS supervised the whole project. All authors have read and approved the manuscript.

## Supplementary Material

Supplemental data

Unedited blot and gel images

Supporting data values

## Version 1 11/05/2024

In-Press Preview

## Version 2 12/20/2024

Electronic publication
