# Supplementary material for: All-in-one AAV-mediated Nrl gene inactivation rescues retinal degeneration in Pde6a mice
Source: JCI Insight. 2024 Dec 20;9(24):e178159. doi: 10.1172/jci.insight.178159 (PMC11665581; doi:10.1172/jci.insight.178159)
Supplement: Supplemental data [file jciinsight-9-178159-s236.pdf]

Supplemental information

All-in-one AAV-mediated *Nrl* gene inactivation rescues retinal degeneration in *Pde6a* mice

Zhiquan Liu[1], Siyu Chen[1], Chien-Hui Lo[1], Qing Wang[1], Yang Sun[1,2]

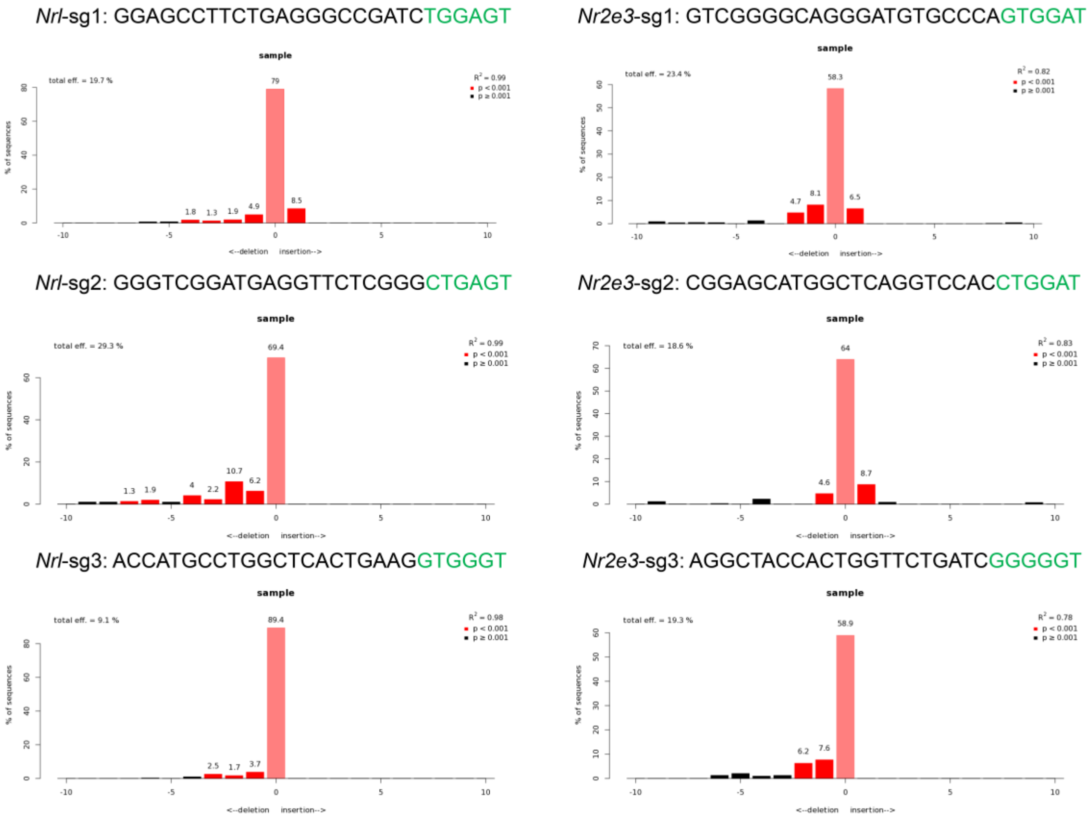

Supplemental Figure 1. Representative indel pattern analyzed by TIDE of the tested sgRNAs targeting *Nrl*/*Nr2e3* using the all-in-one AAV-SaCas9 vector in N2a cells.

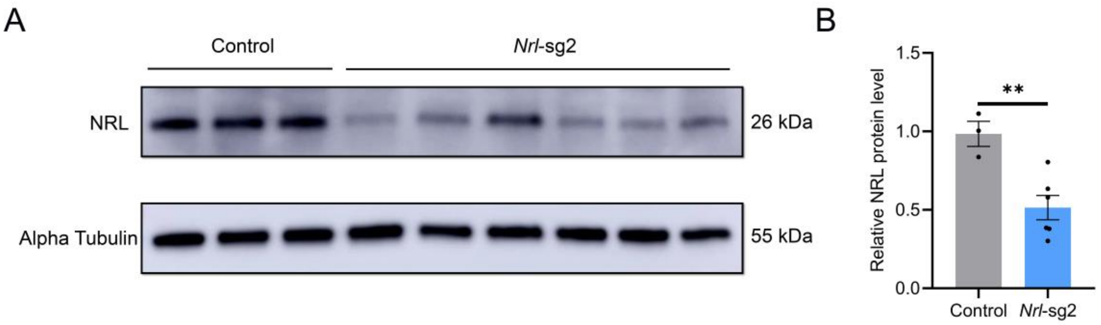

Supplemental Figure 2. Western blot analysis of NRL protein levels. **(A)** Immunoblot analysis of NRL protein expressions in control (n=3 mice) and *Nrl*-sg2 (n=6 mice) treated *Pde6a* mice at P30. **(B)** Quantification of the relative NRL protein level normalized to Alpha tubulin in **(A)**. ***p* < 0.01.

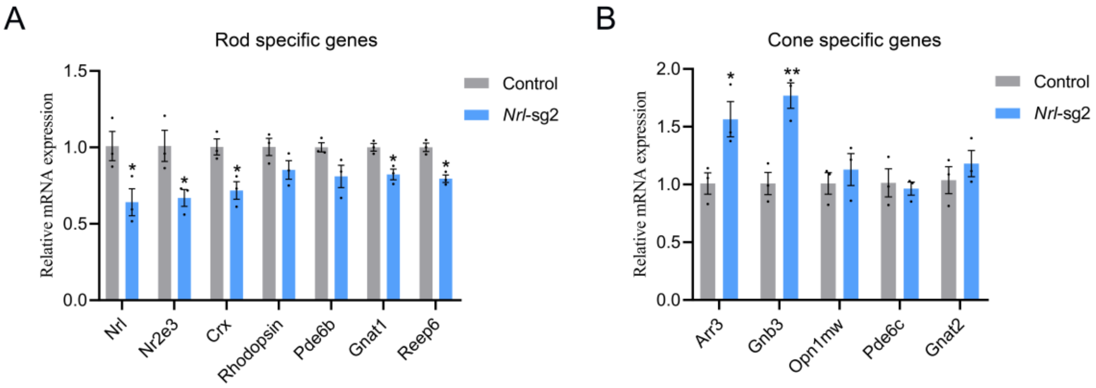

Supplemental Figure 3. The qPCR analysis of rod-specific genes **(A)** and cone-specific genes in control and *Nrl*-sg2 treated *Pde6a* mice at P30 (n=3 mice). RNA from each group were extracted from whole retina tissue. *$p < 0.05$, **$p < 0.01$.

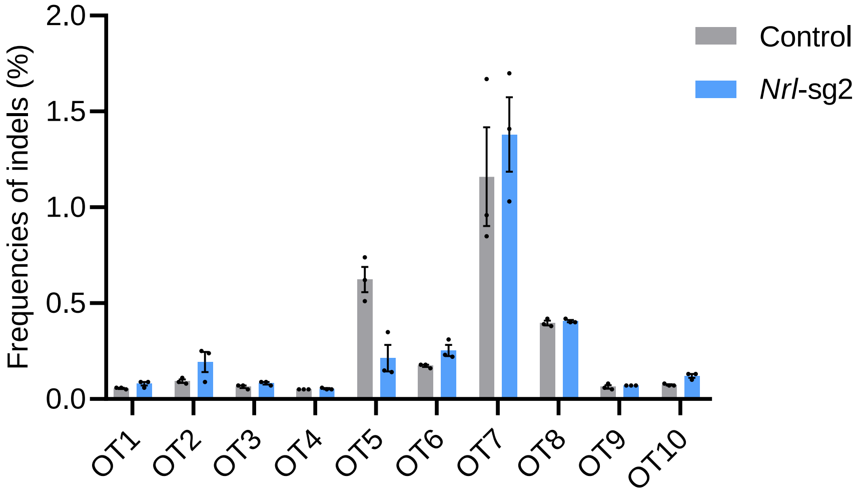

Supplemental Figure 4. Off-target detection in predicted top 10 potential off-target sites by deep sequencing in non-edited control and *Nrl*-sg2 edited mice (n=3 mice).

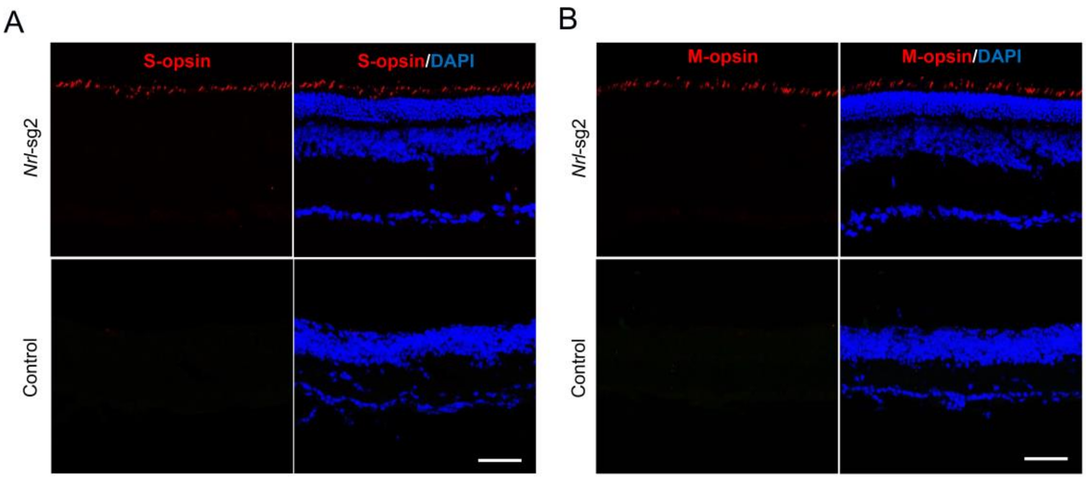

Supplemental Figure 5. Representative immunofluorescence images of S-opsin **(A)** and M-opsin **(B)** in *Nrl*-edited or *Pde6a* control mice at P60. Scale bar, 50 μm.

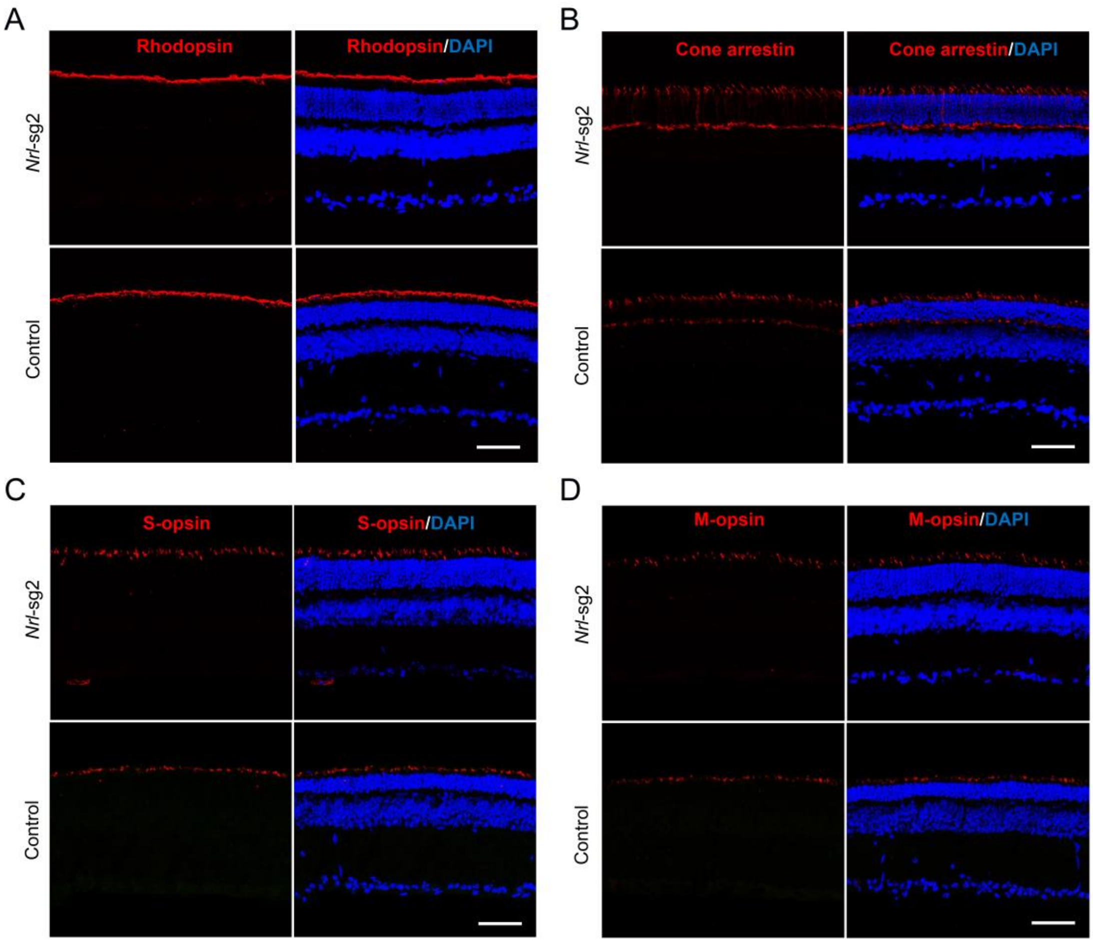

Supplemental Figure 6. Representative immunofluorescence images of Rhodopsin **(A)**, Cone arrestin **(B)**, S-opsin **(C)** and M-opsin **(D)** in *Nrl*-edited or *Pde6a* control mice at P30. Scale bar, 50 μm.

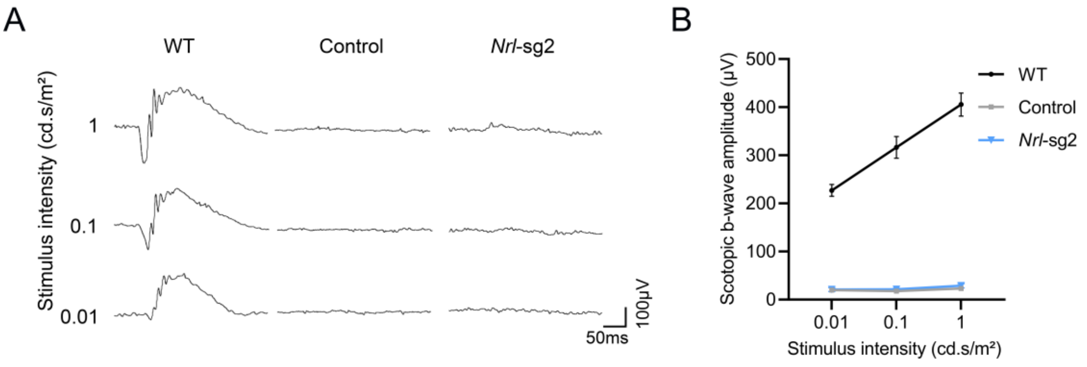

Supplementary Figure 7. Scotopic ERG analysis. **(A)** Representative scotopic ERG responses of WT, *Pde6a* control or *Nrl*-edited mice at P60. The light stimulus intensities are 0.01, 0.1 and 1 cd.s/m^2. **(B)** Quantification of scotopic ERG b wave amplitudes from WT, *Pde6a* control or *Nrl*-edited mice at P60.

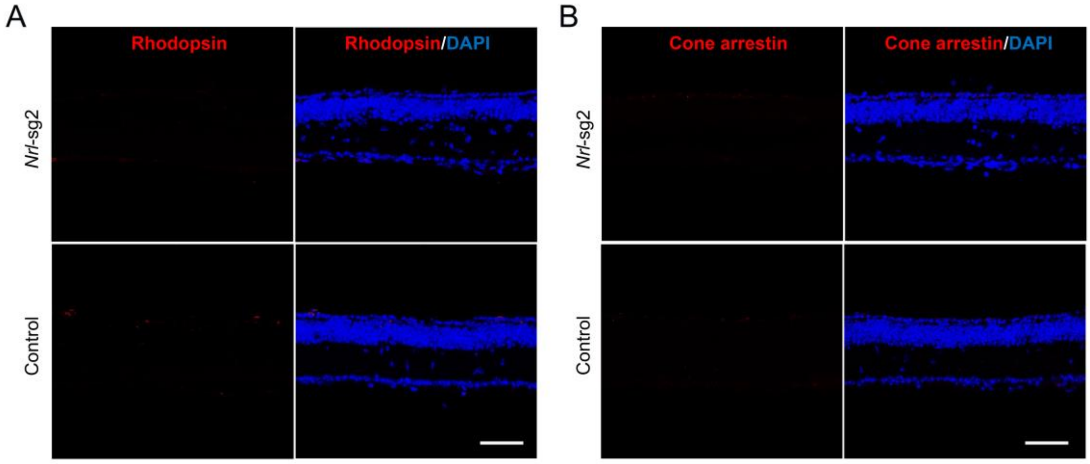

Supplemental Figure 8. Representative immunofluorescence images of Rhodopsin **(A)** and Cone arrestin **(B)** in *Nrl*-edited or *Pde6a* control mice (injected at P30, analyzed at P60). Scale bar, 50 μm.

Supplemental Table 1. The target sgRNAs and synthesized oligos used in this study. Target sequence (black), PAM region (green).

Target site	Sequence (5'-3')	Oligos
Nrl-sg1	GGAGCCTTCTGAGGGCCGATCTGGAGT	F: CACCGGAGCCTTCTGAGGGCCGATC<br>R: AAACGATCGGCCCTCAGAAGGCTCC
Nrl-sg2	GGGTCGGATGAGGTTCTCGGGCTGAGT	F: CACCGGGTCGGATGAGGTTCTCGGG<br>R: AAACCCCGAGAACCTCATCCGACCC
Nrl-sg3	ACCATGCCTGGCTCACTGAAGGTGGGT	F: CACCGCCATGCCTGGCTCACTGAAG<br>R: AAACCTTCAGTGAGCCAGGCATGGC
Nr2e3-sg1	GTCGGGGCAGGGATGTGCCCAGTGGAT	F: CACCGTCGGGGCAGGGATGTGCCCA<br>R: AAACTGGGCACATCCCTGCCCCGAC
Nr2e3-sg2	CGGAGCATGGCTCAGGTCCACCTGGAT	F: CACCGGGAGCATGGCTCAGGTCCAC<br>R: AAACGTGGACCTGAGCCATGCTCCC
Nr2e3-sg3	AGGCTACCACTGGTTCTGATCGGGGGT	F: CACCGGGCTACCACTGGTTCTGATC<br>R: AAACGATCAGAACCAGTGGTAGCCC

Supplemental Table 2. The predicted potential off-target sites of used sgRNAs by Cas-OFFinder.

Target site	Number of mismatches	Number of predicted off-target sites
Nrl-sg1	1	0
	2	0
	3	0
	4	5
Nrl-sg2	1	0
	2	0
	3	1
	4	14
Nrl-sg3	1	0
	2	0
	3	2
	4	30
Nr2e3-sg1	1	0
	2	1
	3	0
	4	2
Nr2e3-sg2	1	0
	2	0
	3	1
	4	8
Nr2e3-sg3	1	1
	2	0
	3	1
	4	6

Supplemental Table 3. Primers used for detecting target site editing in this study.

**Primers**	**Sequence (5'-3')**	**Product size (bp)**
Nrl-F:<br>*Nrl*-R:	GCTGGATCTGTACTCTGGAATC<br>TCCAGGACTTATCCTTCCTCTT	634
Nr2e3-F:<br>*Nr2e3*-R:	CTTCAAGAGGAGTGTGAGAAGG<br>GCTCCAGTTTAGCACAAGTTTC	606
Nrl-deepseq-F:<br>*Nrl*-deepseq-R:	AGGCCTGGAGGAGCTATATT<br>GGACCTTGTTCTGTCTCTTTCC	295

Supplemental Table 4. Primers used for detecting off-target editing in this study. The mismatched nucleotides are shown in lowercase.

Potential off-target site	Number of mismatches	Position	Deep sequencing primer
GGGTaGGAgGAGGT TCTgGGGTTGAGT	3	chr7:+43947618	OT1-F: CCAGCCTGAGCTACAAAGAA<br>OT1-R: CGCTGCCTGAACAAGATAGA
GGcTCtGgTGAGGTT CTCGtGGTGAGT	4	chr8:-47599544	OT2-F: CTGGCTTGGAGGTTGTTACT<br>OT2-R: GGAGGACCACTGGTTTAATAGG
GGGTCaaATGAGGTT aTCaGGGTGAGT	4	chr5:-43039829	OT3-F: CGCAAGCCACAGTTTCTTAAC<br>OT3-R: TATCTCCGGGTAGCAGTGAA
GGGTaGGATGAGGaT gTgGGGAGGAAT	4	chr1:-181502401	OT4-F: GTGGAGAGGCAGATGGTAATAG<br>OT4-R: GCTGTAGTGGGTTACTCAGAAG
GGGTaGGAgGAGGcT CTgGGGTTGGGT	4	chr7:+44231259	OT5-F: TACTTATGATGCTGAGGCAAGAG<br>OT5-R: TGAGCAAGGGCTTCGTATTT
GaGTaGGAgGAGGTT CTgGGGTTGGGT	4	chr7:+44077763	OT6-F: CTCAGAGAAAGCCCAAGAAAGA<br>OT6-R: ACTGACTCTCCTTCCCAGTT
GGGTCtGcTGAGGTT CaCtGGAAGGGT	4	chr7:+120920995	OT7-F: GTAGGGACAGATCACGTTAGGA<br>OT7-R: TCCTGGAACTCACTCTGTAGAC
GGGTCccATGAGGTT CTgGGaATGGAT	4	chr17:-77252409	OT8-F: GAGGTACATGATATGAAACTGACAAAG<br>OT8-R: GACAGAGACAGACACAAAGACA
GGGTgcaATGAGGTT CTaGGGCAGGAT	4	chr14:-14528806	OT9-F: CTGGCCTCCAACTCTGAAAT<br>OT9-R: GCTACCTTATTGCTCTGTCCTC
GGaTCaGATGtGGcTC TCGGGTTGAGT	4	chr19:+24443748	OT10-F: GCAAGTCATCTGGAGTTGGT<br>OT10-R: GGTCCTGTGGTTTATGCTCTT

Supplemental Table 5. The qPCR primers used in this study.

**Gene**	**Sequence (5'-3')**
Nrl	F: CCTTCTGAGGGCCGATCTG<br>R: GACATGCTGGGCTCCTGTC
Nr2e3	F: ACCAGTCCCAGGTGATGCTA<br>R: CTCAAAGATGGGAGCAGGAG
Crx	F: AAACTGAGCTGGGATGCTGT<br>R: TTGTGCCCCCTCAATCTAAC
Rhodopsin	F: TCAGTCTGCATCCCTCCTCT<br>R: CCCAGTTTCCATCCATTTTG
Pde6b	F: GCCGTGTTTCATGGCTTT<br>R: TCCAAAGTTACATTCGATCTTTTT
Gnat1	F: CCCCTCAAATACCGTCCTTT<br>R: GCTGCTGTAGGTCCAAGAGG
Reep6	F: CGGTTACGGGGCCTCTCTA<br>R: CCAGTAGGTTAGCCACACAGT
Arr3	F: AAAGCTCCAACAGCAGACAGA<br>R: TGGACAAAATGATTTATTAGAGTGTTG
Gnb3	F: AAGAAGCAGATTGCTGATGCC<br>R: GTCCCCTTAATGTCCTCCGTG
Opn1mw	F: TCTCTTTGGAAAGAAGGTTGATG<br>R: TGAGAAGGGAGGTAAAACATGG
Pde6c	F: ATCCAAAAGAGCCTCCTTGA<br>R: TTTCCAGGTCAGCAATGGAT
Gnat2	F: AAACCACCCCAAAGCCTAAC<br>R: GAAATAAGCAGGCTCGCATC
Gapdh	F: CGTCCCGTAGACAAAATGGT<br>R: TTGATGGCAACAATCTCCAC
