# Supplementary figures and images for: All-in-one AAV-mediated Nrl gene inactivation rescues retinal degeneration in Pde6a mice
Source: JCI Insight. 2024 Dec 20;9(24):e178159. doi: 10.1172/jci.insight.178159 (PMC11665581; doi:10.1172/jci.insight.178159)

Full unedited gel for Supplemental Figure 2A

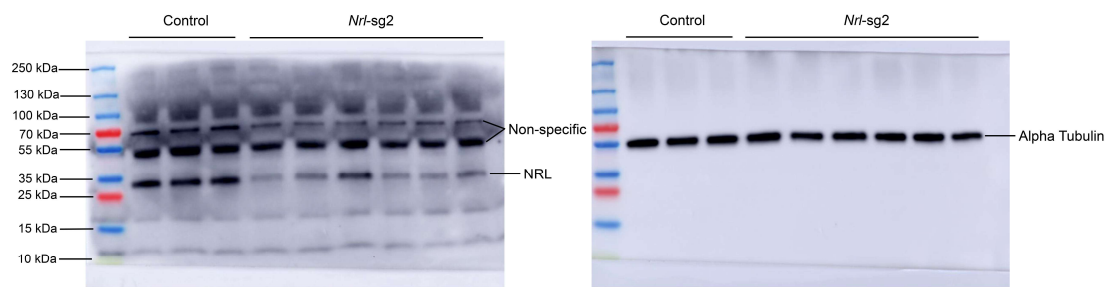

Supplement: Unedited blot and gel images [file jciinsight-9-178159-s237.pdf]
